# Development and nationwide validation of kidney graft injury markers using urinary exosomes and microvesicles (complete English translation of the Japanese version)

**DOI:** 10.1186/s12882-023-03189-z

**Published:** 2023-06-06

**Authors:** Hiroshi Harada, Nobuyuki Fukuzawa, Toyofumi Abe, Ryoichi Imamura, Noriyuki Masaki, Nobuhiro Fujiyama, Shigeru Sato, Shingo Hatakeyama, Kenji Nishimura, Hidefumi Kishikawa, Daiki Iwami, Kiyohiko Hotta, Masayoshi Miura, Kentaro Ide, Michio Nakamura, Akihiro Kosoku, Junji Uchida, Taku Murakami, Takahiro Tsuji

**Affiliations:** 1 Department of Kidney Transplant Surgery, Sapporo City General Hospital, 1-1 Kita 11-jo Nishi 13-chome, Chuou- ku, Sapporo, Hokkaido 060-8604 Japan; 2 Department of Urology, Graduate School of Medicine, Faculty of Medicine, Osaka University, 1 Machikaneyama- cho, Toyonaka, Osaka 560-0043 Japan; 3 Department of Kidney Surgery, Tokyo Women’s Medical University, 8-1 Kawada-cho, Shinjuku-ku, Tokyo, 162- 8666 Japan; 4 Department of Center for Kidney Disease and Transplantation, Akita University Hospital, 44-2 Hiroomote Azahasunuma, Akita, Akita 010-8543 Japan; 5 Department of Urology, Hirosaki University Graduate School of Medicine, 5 Zaifu-cho, Hirosaki, Aomori 036-8562 Japan; 6 Department of Urology, Hyogo Prefectural Nishinomiya Hospital, 13-9 Rokutanji-cho, Nishinomiya, Hyogo Japan; 7 Division of Renal Surgery and Transplantation, Department of Urology, Jichi Medical University, 3311-1, Yakushiji, Shimotsuke, Tochigi 329-0498 Japan; 8 Department of Renal and Genitourinary Surgery, Graduate School of Medicine, Hokkaido University, Kita 15-jo Nishi 7-chome, Kita-ku, Sapporo, Hokkaido 060-8638 Japan; 9 Department of Kidney Transplant Surgery, Sapporo Hokuyu Hospital, 5-1 Higashi-sapporo 6-jo 6-chome, Shiroishi- ku, Sapporo, Hokkaido 003-0006 Japan; 10 Department of Gastroenterological and Transplant Surgery, Graduate School of Biochemical and Health Sciences, Hiroshima University, 1-2-3 Kasumi, Minami-ku, Hiroshima, 734-8553 Japan; 11 Department of Transplant Surgery, Tokai University School of Medicine, 143 Shimokasuya, Isehara, Kanagawa 259-1193 Japan; 12 Department of Urology, Osaka Metropolitan University Graduate School of Medicine, 1-4-3, Asahi-Machi, Abeno-ku, Osaka, Osaka 545- 8585 Japan; 13 R&D Center, Hitachi Chemical Co. America, Ltd. 1003 Health Sciences Road, Irvine, CA 92617 USA; 14 Department of Pathology, Sapporo City General Hospital, 1-1 Kita 11-jo Nishi 13-chome, Chuou-ku, Sapporo, Hokkaido 060-8604 Japan; 15 Harada Urological Clinic, 4F Hokuyaku Bldg., 1-1 Kita 11-jo Nishi 14-chome, Chuou-ku, Sapporo, Hokkaido 060-0011 Japan

**Keywords:** Kidney transplant, Biomarker, Urine, Extracellular vesicle, mRNA

## Abstract

**Background:**

Non-invasive, prompt, and proper detection tools for kidney graft injuries (KGIs) are awaited to ensure graft longevity. We screened diagnostic biomarkers for KGIs following kidney transplantation using extracellular vesicles (EVs; exosomes and microvesicles) from the urine samples of patients.

**Methods:**

One hundred and twenty-seven kidney recipients at 11 Japanese institutions were enrolled in this study; urine samples were obtained prior to protocol/episode biopsies. EVs were isolated from urine samples, and EV RNA markers were assayed using quantitative reverse transcription polymerase chain reaction. Diagnostic performance of EV RNA markers and diagnostic formulas comprising these markers were evaluated by comparison with the corresponding pathological diagnoses.

**Results:**

EV CXCL9, CXCL10, and UMOD were elevated in T-cell-mediated rejection samples compared with other KGI samples, while SPNS2 was elevated in chronic antibody-mediated rejection (cABMR) samples. A diagnostic formula developed through Sparse Logistic Regression analysis using EV RNA markers allowed us to accurately (with an area under the receiver operator characteristic curve [AUC] of 0.875) distinguish cABMR from other KGI samples. EV B4GALT1 and SPNS2 were also elevated in cABMR, and a diagnostic formula using these markers was able to distinguish between cABMR and chronic calcineurin toxicity accurately (AUC 0.886). In interstitial fibrosis and tubular atrophy (IFTA) urine samples and those with high Banff chronicity score sums (BChS), POTEM levels may reflect disease severity, and diagnostic formulas using POTEM detected IFTA (AUC 0.830) and high BChS (AUC 0.850).

**Conclusions:**

KGIs could be diagnosed with urinary EV mRNA analysis with relatively high accuracy.

## Introduction

The kidney transplant is becoming increasingly successful worldwide and in Japan [2]; however, kidney grafts can be affected by ischemic reperfusion injury due to transplantation, and the effect of allograft rejection cannot be completely eliminated. Moreover, drug-induced kidney injury by immunosuppressants, chronic ischemic organ damage due to arterial disease, and original disease of the kidney can occur. Thus, kidney grafts are prone to losing their function gradually due to kidney graft injury (KGI) [3]. A prompt and correct diagnosis, as well as proper treatment of the precious kidney grafts are thus necessary to ensure their continued success.

KGIs are first suspected by clinical findings, urine or blood chemical analyses, and imaging results; the definitive diagnosis is made on histology. Kidney allograft biopsies can now be performed relatively safely following the development of improved technology; however, the procedure is still somewhat invasive and not consistently feasible. Thus, biomarkers that can distinguish between the different types of KGI with high accuracy and low invasiveness are yet to be discovered [4–8].

Recently discovered extracellular vesicles (EVs; exosome and microvesicle) in urine are gaining attention as a source of biomarkers, and we have previously investigated a diagnostic system of KGI using quantitative mRNA analysis of urine EVs [9–13]. We have also presented the results of employing a quantitative reverse transcription polymerase chain reaction (RT-PCR) (qPCR) method for the newly developed correcting and extracting system of mRNA among EVs from urine samples of kidney recipients [14]. This multicenter national study was supported by a grant from the Japanese Society for Clinical Renal Transplantation.

## Materials and methods

### Security of ethicality

This study was performed according to the Helsinki Declaration and complied with both the Ethical Guidelines for Medical and Biological Research Involving Human Subjects and the Ethical Guidelines of Japanese Society for Clinical Renal Transplantation. The ethical committee of each participating facility (Osaka University Graduate School of Medicine, Tokyo Women’s Medical University, Akita University Graduate School of Medicine, Hirosaki University Graduate School of Medicine^,^ Hyogo Prefectural Nishinomiya Hospital, Graduate School of Medicine, Hokkaido University, Sapporo Hokuyu Hospital, Graduate School of Biochemical and Health Sciences, Hiroshima University, Tokai University School of Medicine, and Osaka City University Graduate School of Medicine) approved the study in accordance with the approval of the ethical committee of the principal institution, Sapporo City General Hospital (Development and nationwide validation of kidney graft injury markers using urinary exosome and microvesicle: H28-053-319).

### Collection of urine samples and management

Urine samples were collected via voiding or a Foley catheter from 127 patients. Kidney graft pathological diagnoses were made for these patients using episode or non-episode protocol biopsy (Table 1) in the 11 kidney transplant facilities in Japan including Sapporo City General Hospital and stored in 15 or 50 mL specimen tubes. All specimens were stored in a deep freezer (-80° C) at each facility and transferred to Sapporo City General Hospital where it was also stored at -80° C. All specimens were then shipped to the collaborating facility in CA, USA with dry ice below − 80 °C and stored at -80° C until the assay.

### Pathological diagnosis

Kidney biopsy preparations from each patient were provided together with the urine samples, and a central pathologist (T.T.) reviewed all preparations by the Banff criteria [3] according to clinical course and pathological diagnoses at each facility. The final pathological diagnoses were classified as stable recovery without any abnormality (Stable recovery), Borderline change, T-cell-mediated rejection (TCMR), acute antibody-mediated rejection (aABMR), chronic-antibody mediated rejection (cABMR), acute calcineurin inhibitor nephrotoxicity (aCNIT), chronic calcineurin inhibitor nephrotoxicity (cCNIT), or interstitial fibrosis/tubular atrophy (IFTA, grade I-III). Banff chronicity score sum (BChs) was calculated according to the Banff chronicity score (cg, ci, ct, cv) and defined as high BChs if > 3 and as low BChs if < 2. The results of pathological diagnoses are listed in Table 1.

### Urine extracellular vesicle recovery

All stored urine samples were thawed for 5 min (15 min in the case of 50 mL samples) in a warm (37° C) water bath, and supernatants were collected after 800xG centrifugation. Next, we performed EV recovery and extracted mRNA from 10 ml of supernatant using ExoComplete Kit (Hitachi Chemical Diagnostics, CA, USA) according to the instruction manual [8].

### Candidate marker mRNA assay using quantitative RT-PCR

Thirty-nine genes were analyzed by quantitative RT-qPCR as previously described [8]. These included candidate genes that were utilized in previous research [14], those that were nominated by a next-generation sequencer (NGS), and housekeeping genes (ACTB, AIF1, ALDOB, ANXA1, B4GALT1, BTN3A3, CCL5, CD3E, CD48, CD59, CRYBG2, CXCL9, CXCL10, DUOX2, EMP1, EPHA2, EPS8L1, FCGBP, GAPDH, GZMB, HAVCR1, HOXB13, HSPB8, KLK2, KLK3, MAL, MAP3K9, PITX1, POTEM, PRF1, PRSS8, RDH10, SLC12A1, SLC45A3, SLC6A6, SPDEF, SPNS2, TMEM127, and UMOD). The threshold cycle (Ct) for each gene was standardized according to the delta Ct method using the Ct value of GAPDH as a reference. To ensure the quality and accuracy of further analyses, samples with a Ct value of GAPDH > 30 were excluded (15 of 150 samples). R was used for data analysis, and analyses for which *p* was < 0.05 were considered statistically significant using the Welch *t*-test. Sparse Logistic Regression analysis was used to calculate each diagnostic formula as previously reported [10]. Briefly, Sparse logistic regression analysis was conducted using glmnet with 10-fold cross-validation and 5000 bootstrap re-sampling on the gene expression values of the 39 genes. The performance of each formula was then evaluated using the area under the curve (AUC) of receiver operator characteristic curve analysis, and the sensitivity and accuracy were calculated from the point closest to the top left corner.

## Results

### Patient background and pathological diagnosis

Background and demographic information for the 127 patients enrolled in this study are shown in Table 1. Non-episode protocol graft biopsy was performed in 58 patients, and episode biopsies for suspicious graft injuries such as graft rejection were performed in 69 patients. The pathological diagnoses included 38 Stable recoveries, 13 TCMR, 8 aABMR, 32 cABMR, 3 aCNIT, and 19 cCNIT. IFTA was seen in 34 patients, and the grades were IFTA I in 17 of them, II in 13, and III in 4. The BChS was < 2 in 53 patients, 2 or 3 in 37, and > 3 in 35.

**Table 1:** Patient background characteristics

Items	Number of numerical values
Recipient’s sex (male/female) (number)	48/79
Age at transplantation (median – range) (age)	44 (10–73)
Unknown case (number)	2
Original disease (number)	
Chronic glomerulonephritis	29
IgA nephropathy・IgA vasculitis	40
Focal segmental glomerulosclerosis・nephrotic syndrome	4
Diabetic nephropathy	10
Nephrosclerosis	7
Autosomal dominant polycystic kidney disease	4
Congenital anomaly of kidney and urinary tract	8
Others	14
Unknown etiology	11
Donor type (live/cadaver) (number)	112/15
Relation to recipient (number)	
Parent	59
Sibling	8
Offspring	2
Other relative	1
Spouse	42
Cadaveric donor	15
Donor age (median – range) (age)	56 (25–82)
Blood type (compatible・incompatible) (number)	100/27
Induction immunosuppression - calcineurin inhibitor (number)	
Tacrolimus	110
Ciclosporin	16
Sparing	1
Period of kidney graft biopsy (median - range) (month)	24 (0.067–485)
Reason for biopsy (including repeated case) (case)	
Acute rejection suspected	47
Chronic rejection suspected	10
Proteinuria	8
BK-polyoma virus nephropathy suspected	4
Calcineurin inhibitor nephropathy suspected	1
Protocol biopsy	58
Pathological diagnosis (including repeated case) (case)	
Stable recovery	38
Borderline change	6
T-cell mediated rejection	13
Acute antibody-mediated rejection	8
Chronic antibody-mediated rejection	32
Acute calcineurin inhibitor nephrotoxicity	3
Chronic calcineurin inhibitor nephrotoxicity	19
Interstitial fibrosis/tubular atrophy Grade I	17
Interstitial fibrosis/tubular atrophy Grade II	13
Interstitial fibrosis/tubular atrophy Grade III	4
Banff chronicity score sum < 2	53
Banff chronicity score sum 2–3	37
Banff chronicity score sum > 3	35

### Gene analysis of graft injury by quantitative RT-PCR

#### Discrimination of rejection type

**Fig. 1:**
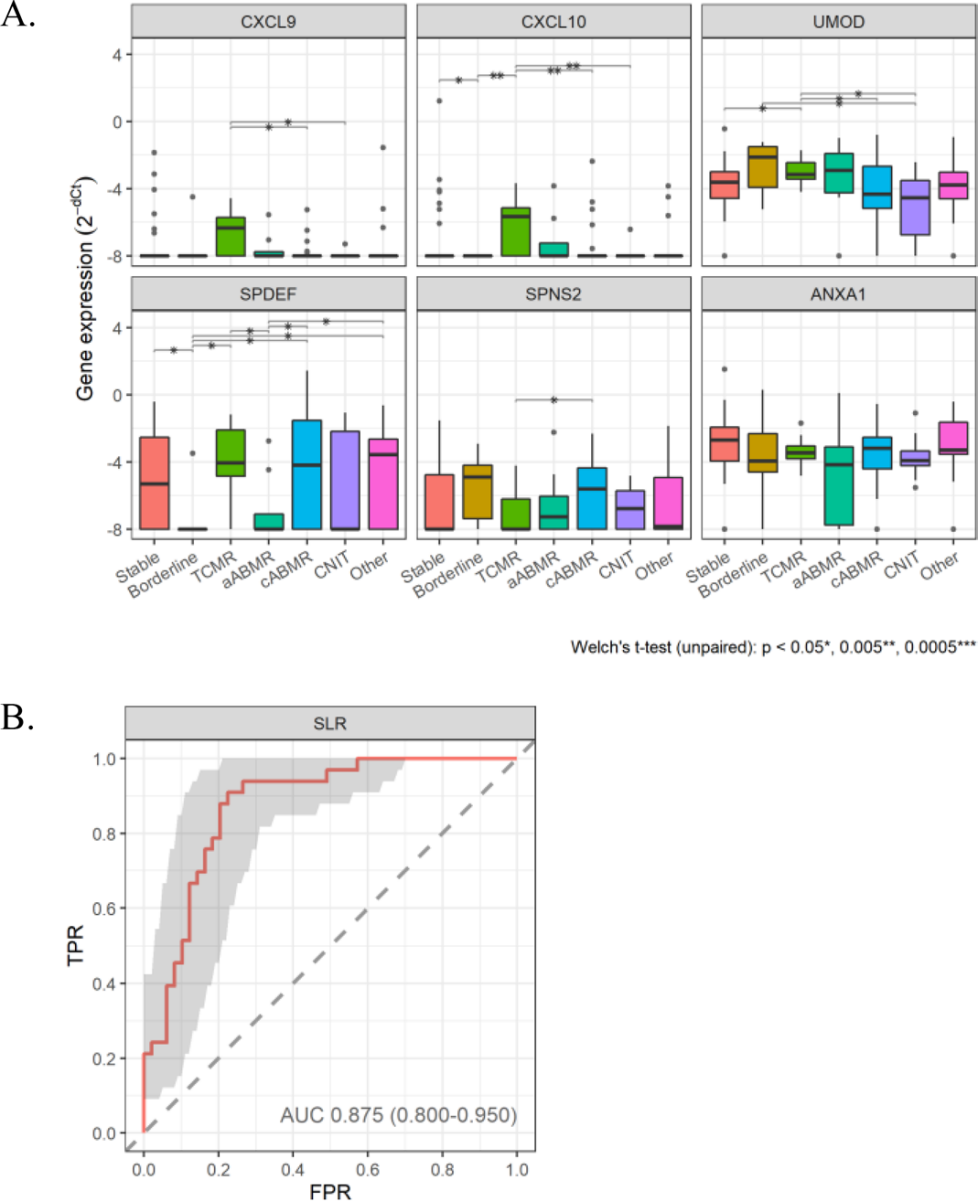
RT-qPCR analysis of candidate genes (CXCL9, CXCL10, UMOD, SPDEF, SPNS2, and ANXA1) for all kidney graft injuries (KGIs), Borderline, Borderline change; CNIT, aCNIT+cCNIT (**A**) and evaluation of the performance of the diagnostic formula in distinguishing cABMR from other KGIs by Sparse logistic regression (SLR) analysis (**B**)

The results of qPCR analyses are shown in Fig. 1A. CXCL9, CXCL10, UMOD, SPDEF, and SPNS2 were differentially expressed among graft rejections. The genetic expression patterns of CXCL9 and CXCL10 were notable; they were elevated in TCMR but not in antibody-mediated rejections, supporting previous results [7, 15, 16]. Elevation of UMOD, which is reportedly an important EV biomarker for disease progression toward diabetic kidney disease among patients with type II diabetes [12], was also seen in patients with TCMR in the present study and may thus be a potential biomarker for TCMR.

In contrast, the detection of cABMR was more challenging. Elevation of SPNS2 and SPDEF expression was observed in cABMR, although this was also the case for other KGIs. Previously, we reported that ANXA1 increased in TCMR and cABMR [14]; however, it was not elevated in cABMR or the other KGIs in the present study. A single biomarker alone is unlikely to distinguish between graft rejection types sensitively and precisely. However, establishing a diagnostic formula could improve the diagnostic power using logistic regression analysis as previously reported [10]. Sparse logistic regression analysis was conducted using glmnet with 10-fold cross-validation and 5000 bootstrap re-sampling on the gene expression values of the 39 genes. The best diagnostic formula selected by SLR was able to distinguish cABMR from other KGIs with an AUC of 0.875 (sensitivity 87.9%, accuracy 79.6%) (Fig. 1B).

#### Discrimination between chronic active antibody-mediated rejection and chronic calcineurin inhibitor toxicity

**Fig. 2:**
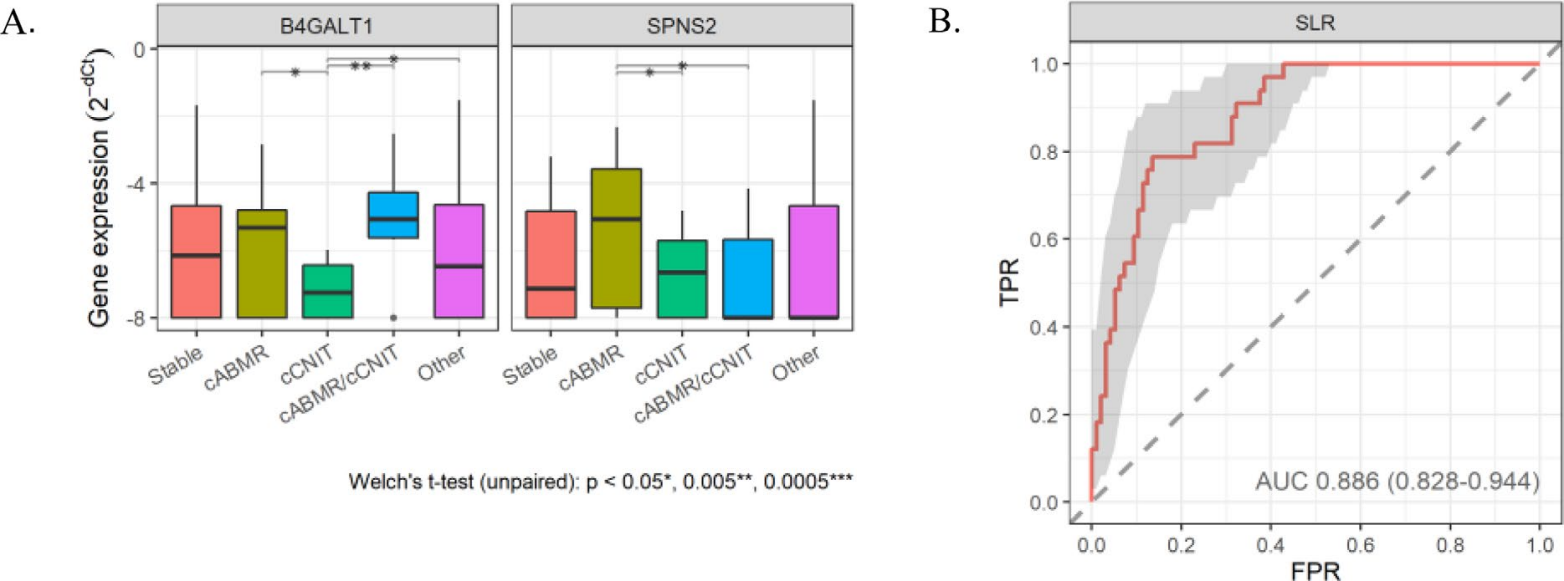
RT-qPCR analysis of candidate genes (B4GALT1 and SPNS2) to differentiate between cABMR and cCNIT (**A**) and evaluation of the performance of the diagnostic formula in distinguishing cABMR from cCNIT by Sparse logistic regression (SLR) analysis (**B**)

cCNIT is not graft rejection; however, it is difficult to clinically distinguish it from cABMR. Thus, we investigated ways to distinguish between cABMR and cCNIT by EV RNA. B4GALT1 and SPNS2 gene expressions were enhanced in cABMR but suppressed in cCNIT (Fig. 2A). The established diagnostic formula by SLR analysis improved the diagnostic performance up to an AUC of 0.886 (sensitivity 78.8%, accuracy 86.5%) compared to an AUC of 0.64 by single gene analysis (Fig. 2B).

#### Determination of interstitial fibrosis and tubular atrophy severity.

**Fig. 3:**
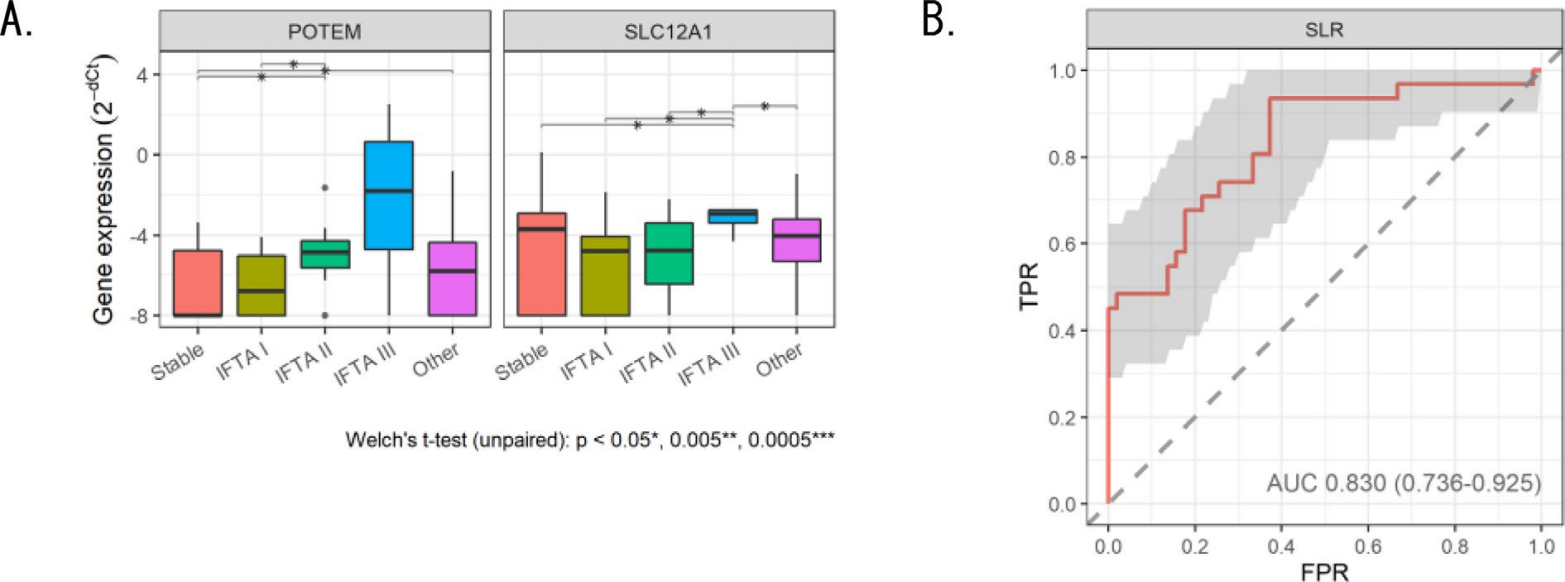
RT-qPCR analysis of candidate genes (POTEM or SLC12A1) to judge the severity of IFTA (**A**) and evaluation of the performance of the diagnostic formula by Sparse logistic regression (SLR) analysis (**B**)

Next, we performed EV-mRNA analysis to evaluate the severity of IFTA and test whether EV-RNA analysis would be complementary to diagnosis by graft biopsy pathology (Fig. 3A). POTEM and SLC12A1 gene expressions were elevated according to IFTA severity. The formula by SLR analysis determined IFTA severity with an AUC of 0.830 (sensitivity 71.0%, accuracy 78.4%) (Fig. 3B), demonstrating its utility as a diagnostic method complementary to graft biopsy pathology.

#### Determination of the severity of chronic kidney graft injury

**Fig. 4:**
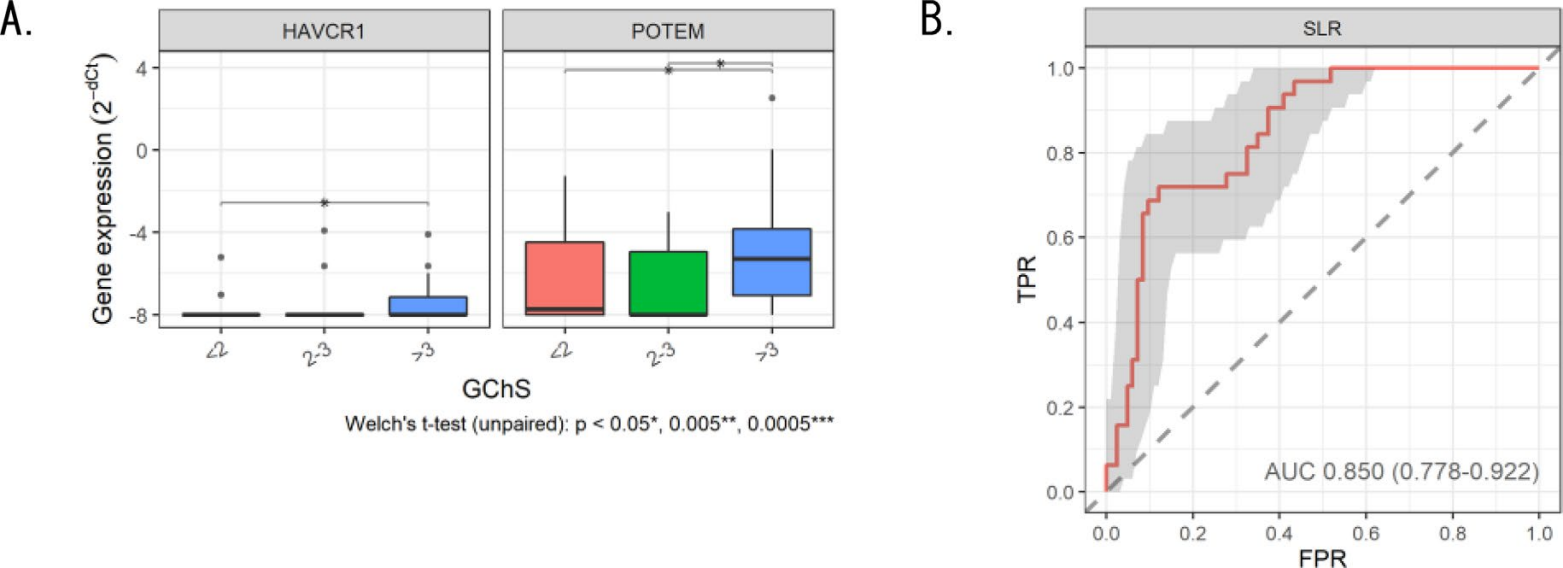
RT-qPCR analysis of candidate genes (HACR1 or POTEM) to judge the severity of chronic graft injury according to BChS (**A**) and evaluation of the performance of the diagnostic formula in distinguishing between high and low BChS by Sparse logistic regression (SLR) analysis (**B**)

The BChS scoring system reflects the severity of chronic KGI and requires graft biopsy pathology, similar to the assessment of IFTA severity. High BChS indicates a high risk of graft loss [17]. In the correlation analysis between BChS and EV-RNA expression levels, BChS was correlated with HAVCR1 and POTEM (Fig. 4A). Since we generated the diagnostic formula by SLR analysis for all 39 genes, BChS was distinguished with an AUC of 0.850 (sensitivity 71.9%, accuracy 88.0%) (Fig. 4B). Finally, patients with graft loss were not enrolled in this study; therefore, the correlation between the presented results and graft loss should be verified.

## Discussion

Kidney transplantation is the standard renal replacement therapy. Despite the requirement of immunosuppressants to suppress any immunological reaction against alloimmunity, kidney transplantation improves the life expectancy and quality of life of patients with end-stage kidney disease as compared to dialysis therapies. Although the outcome of kidney transplantation has been improving following the development of immunosuppressants and the increased understanding of proper management for graft rejection, kidney grafts tend to lose their function due to allograft rejections, as well as problems such as recurrence of the original disease, toxicity of immunosuppressants, development of metabolic disorders, and glomerular overload [2]. Improving survival of kidney grafts is thus a challenge and an unsolved issue.

The diagnosis of graft injury (including graft rejection) relies on clinical manifestations such as a decrease in urine volume and fever, urine and blood analyses, blood chemical analysis, and radiographic evaluation such as ultrasonography, computed tomography, or radioisotope imaging. However, the definitive diagnosis is still made by graft pathology [3, 4]. A graft biopsy can generally be performed safely; however, there are risks for patients in the early period after kidney transplantation and for those taking antithrombotic agents because of slight invasiveness. Moreover, the final diagnosis takes several days. Thus, there is a need for a non-invasive biomarker assay that can yield a correct diagnosis of graft injuries with a comparable performance to histology. An assay of chemical substances such as neutrophil gelatinase-associated lipocalin [6, 18] and liver-type fatty acid-binding protein [19], which have been proposed to be enhanced in tubular injury; however, a diagnostic modality that can detect several types of graft injury is ideal. We focused on exosomes in patient urine in this study. Exosomes are microvesicles discharged from cells and include cell membrane components, protein, DNA, mRNA, and miRNA. Additionally, exosomes have been focused on as an information source since the late 1990s [20]. The intercellular signal from renal injury and lymphocytes is included. Strictly, the sizes of exosomes and microvesicles are 50–100 nm and 100–1000 nm, respectively, but they are often collectively referred to as EVs [20]. EVs are located in blood or fluids such as bile or ascites [13] and can be recovered from any part of the body. They are an ideal biomarker source for investigating kidney or urinary tract disease [5, 21, 22] because EVs from urine can be recovered non-invasively. The efficacy of EV evaluation by RT-PCR in nephritis [11] diabetic kidney disease [12], and bladder cancer [10] has been previously established. Furthermore, EVs are generally retrieved by an ultracentrifugation method; however, this procedure is complicated, and yields limited measurable samples [23]. As an alternative, we explored the seamless assay system for recovery of EV, extraction of mRNA, and generation of cDNA, establishing a protocol for the rapid management of multiple samples [9]. A critical step during the mRNA assay is preventing damage by RNase among urine samples contaminated in recovery or storage. Thus far, we have recovered urine samples by way of ordinal sample handling for urinalysis and consecutive freezing preservation within a few hours, yielding RNA that was successfully measured. This may be because EVs are covered with cellular lipid membranes; RNA is thus protected from temperature changes and RNase, preventing its degradation. Consequently, EVs are an ideal source of information [20].

In this study, we evaluated KGI using the measurement of mRNA obtained from urine EVs after having previously introduced the usefulness of a single gene, ANXA1, in the detection of graft injury in a single center analysis of kidney injury model [14]. Subsequently, a nationwide survey including the search of candidate genes by NGS was developed to verify this result.

Here, 39 candidate genes selected based on our preparation study were analyzed using qPCR from 127 patients. CXCL9, CXCL10, SPDEF, SPNS2, and UMOD showed statistical differences between some graft rejection types. Among these, CXCL9/CXCL10 and UMOD were shown to be significant biomarkers of TCMR, as their expression showed robust enhancements in samples from patients with TCMR; in contrast, there was no increase in the expression of these genes in samples from patients with antibody-mediated rejection. Previous literature has stated that the chemokines CXCL9 and CXCL10 are significant biomarkers for detecting allograft rejection in animal models and a clinical multiple-institute study. Our present study clearly supports these results [7, 8, 15, 16]. In this study, the detection of TCMR by single genes other than CXCL9 or CXCL10 was difficult; however, a combination of multiple candidate mRNA generated reliable diagnostic formula and became the promising biomarker instead of graft biopsy and pathology in the diagnosis of KGI. For example, we also determined that UMOD can be an alternative biomarker for TCMR detection. UMOD is a gene-encoding uromodulin, also called Tamm-Horsfall protein. Uromodulin, a kidney-specific protein located in the medullary thick ascending limb of the loop of Henle, is reportedly a predictor of tissue injury in patients with anti-neutrophil cytoplasmic antibody-related nephritis [24]. Moreover, UMOD expression in urine EVs is a predictive biomarker of the development of diabetic kidney disease in patients with type 2 diabetes [12].

B4GALT1 expression was increased in cABMR but decreased in cCNIT. Both KGIs induce gradual arteriole stenosis and consecutive tissue injury as a result of chronic ischemic changes. B4GALT4 is a promising gene biomarker for distinguishing between these two events and has critical significance given the contrary management of immunosuppressant dosing for these conditions. B4GALT1 is a gene encoding glycosyltransferase and influences B cell activation [25] and has been used as a predictive marker for disease progression and prognosis in malignancy [26]. The relationship between B4GALT4 and kidney injuries has not been well studied. In the present study, SPNS2 was also nominated as a biomarker gene and has similar expression patterns to B4GALT1. SPNS2 plays a role in anti-fibrotic and anti-inflammatory processes in human kidney gene tissue [27].

SLC12A1 was identified as a candidate marker for reflecting the severity of IFTA by qPCR analysis. NKCC exists on the cell surface and has two variants, NKCC1 and NKCC2; NKCC2 is expressed only in kidney tissue and encoded by SLC12A1 [28]. However, the role of NKCC in graft fibrosis and tubular atrophy is not understood.

Finally, POTEM and HAVCR1 were nominated as the candidate biomarkers for the detection of BChS, which is supposed to correlate with graft loss. HAVCR1, also called TIM-1, is a known biomarker of kidney injury and has been proven to be a candidate marker for chronic KGI. While POTEM was also identified as a candidate biomarker, further study is needed regarding its mechanism of involvement in the progression of chronic graft damage.

## Limitations

The results of the present study were obtained from transplant institutions in Japan; however, the sample size was limited. In addition, the recent progress of immunosuppressants along with a thorough understanding of the mechanism of graft rejection and graft pathology, lessen the probability of acute cellular rejection, making the distribution of KGI unequable. Moreover, immunosuppression protocol and follow-up policy were carried out differently by individual facilities. Lastly, donor type was skewed to living donors because of the extreme shortage of deceased organ donors in our country.

## Conclusions

This study focused on urine EVs as a biomarker source and developed a diagnostic modality for graft injury after kidney transplantation using qPCR of mRNA obtained from EVs. This modality was able to incorporate further specific gene explorations beyond those presented here. Moreover, by calculating the diagnostic formula using multiple gene combinations, detected graft injury was detected more accurately. The present study was a nationwide, multicenter study; however, further studies with larger sample sizes are required to validate its results. This study presents the groundwork for the future development of solutions for tough KGI, cABMR, and graft fibrosis. To promote this development, an upcoming prospective long-term study is schemed to ensure graft longevity.

## Data Availability

All data generated during this study are included in this article. Further inquiries can be directed to the corresponding author.

## Abbreviations

KGI: Kidney graft injury
EV: Exosome and microvesicle
RT-PCR: Reverse transcription polymerase chain reaction
qPCR: Quantitative transcription polymerase chain reaction
TCMR: T-cell mediated rejection
aABMR: Acute antibody-mediated rejection
cABMR: Chronic antibody-mediated rejection
aCNIT: Acute calcineurin inhibitor nephrotoxicity
cCNIT: Chronic calcineurin inhibitor nephrotoxicity
IFTA: Interstitial fibrosis and tubular atrophy
BChs: Banff chronicity score sums
NGS: Next generation sequencer
Ct: Threshold cycle
AUC: Area under the curve
